# Prognostic Role of Clinicopathological Characteristics and Serum Markers in Metastatic Melanoma Patients Treated with BRAF and MEK Inhibitors

**DOI:** 10.3390/cancers16172981

**Published:** 2024-08-27

**Authors:** Eszter Anna Janka, Imre Lőrinc Szabó, Sándor Kollár, Tünde Toka-Farkas, Beatrix Ványai, Tünde Várvölgyi, Anikó Kapitány, Hibah Shabu, Andrea Szegedi, Gabriella Emri

**Affiliations:** 1 Department of Dermatology, MTA Centre of Excellence, Faculty of Medicine, University of Debrecen, 4032 Debrecen, Hungary; janka.eszter@med.unideb.hu (E.A.J.); szabo.imre.lorinc@med.unideb.hu (I.L.S.); toka-farkas.tunde@med.unideb.hu (T.T.-F.); vanyai.beatrix@med.unideb.hu (B.V.); varvolgyi.tunde@med.unideb.hu (T.V.); kapitany.aniko@med.unideb.hu (A.K.); hibahshabu@gmail.com (H.S.); aszegedi@med.unideb.hu (A.S.); 2 HUN-REN-UD Allergology Research Group, University of Debrecen, 4032 Debrecen, Hungary; 3 Institute of Pathology, Faculty of Medicine, University of Debrecen, 4032 Debrecen, Hungary; dr.kollar.sandor@gmail.com; 4 Doctoral School of Health Sciences, University of Debrecen, 4032 Debrecen, Hungary

**Keywords:** metastatic melanoma, prognostic factors, targeted therapy, Cox proportional hazard models, ROC curve

## Abstract

**Simple Summary:**

Studies analyzing specific treatment-related prognostic factors can provide important information about disease biology and improve the use of biomarkers to optimize treatment decisions. The aim of our retrospective study was to determine the prognostic performance of clinicopathological factors and blood biomarkers in patients with unresectable metastatic melanoma treated with BRAF and MEK inhibitors. A total of 199 patients were included in the multivariate analysis of the risk of progression and death. We found that primary tumor localization on the limbs, Clark invasion level V, M1c stage or M1d stage at the start of therapy, and elevated baseline serum S100B level were independently and significantly associated with poor outcomes. The present study suggests that clinicopathological factors, including primary tumor characteristics and stage of metastatic disease, as well as serum markers may provide information on the probability of survival in patients with advanced melanoma treated with BRAF and MEK inhibitors.

**Abstract:**

Prognostic studies can provide important information about disease biology and improve the use of biomarkers to optimize treatment decisions. Methods: A total of 199 patients with advanced melanoma treated with BRAF + MEK inhibitors were included in our single-center retrospective study. We analyzed the risk of progression and death using multivariate Cox proportional hazard models. The predictive effect of prognostic factors on progression-free survival (PFS) was evaluated in ROC analysis. Results: We found that primary tumor localization, Clark level, pT category, baseline M stage and baseline serum S100B are independent and significant prognostic factors for PFS. The discriminative power of the combination of these factors was excellent for predicting 18 month PFS (AUC 0.822 [95% CI 0.727; 0.916], *p* < 0.001). Primary tumor localization on the extremities, Clark level V, baseline M1c stage or M1d stage, and elevated baseline serum S100B and LDH levels were independently and significantly associated with unfavorable overall survival (OS). Conclusion: Baseline M stage and serum S100B appear to be independent prognostic factors for both PFS and OS in melanoma patients treated with BRAF + MEK inhibitors. We newly identified significant and independent prognostic effects of primary tumor localization and Clark level on survival that warrant further investigation.

## 1. Introduction

Cutaneous melanoma has a high risk of metastasis, and although the majority of cases are diagnosed with low tumor thickness, the incidence of melanoma and thus the number of metastatic cases is increasing [1]. Due to diagnostic and therapeutic innovations, the outcome of metastatic melanoma has greatly improved over the past decade [2,3]. In patients with stage IV melanoma, immune checkpoint inhibitors are the preferred first-line treatment regardless of BRAF mutational status, and advanced melanoma patients carrying BRAF V600 mutation can also benefit from BRAF + MEK inhibitor treatment in certain cases [4]. In a meta-analysis with an average BRAF mutation prevalence of 47.8% in the included melanoma studies, the risk of death was 1.7 times higher in patients with BRAF mutant melanoma than in patients with BRAF wild-type melanoma [5]. BRAF mutation has been reported to be associated with an increased risk of developing liver and brain metastases [6]. Targeted therapy can rapidly reduce tumor burden even in patients with high serum LDH levels, high tumor burden, aggressive disease, and symptomatic brain metastases, but loss of efficacy due to acquired drug resistance is common [2,3,4,7,8]. Importantly, the clinical outcome of patients with metastatic melanoma is highly dependent on the efficacy of first-line treatment [9,10,11,12]. Clinical trial data suggest that first-line anti-PD-1 + anti-CTLA-4 combination therapy results in better survival rates in patients with BRAF V600 mutant metastatic melanoma than first-line BRAF + MEK inhibitor combination therapy [11,12]. However, data from other cohorts show that survival of patients receiving first-line BRAF + MEK inhibitor therapy is comparable to that of patients receiving first-line immune checkpoint inhibitors when second-line immunotherapy is feasible after BRAF + MEK inhibitor therapy [9,10]. Furthermore, patients in complete remission with first-line targeted therapy have a high chance of long-term survival [7,8]. Comparison of baseline genomic features detected in BRAF V600 mutant melanoma samples from advanced melanoma patients who differentially responded to BRAF + MEK inhibitor therapy is a promising research approach to better understand tumor response and therapeutic resistance and to optimize treatment selection [13]. However, readily available prognostic biomarkers can more directly lead to clinical benefits if there is a difference in the success of the given therapy in the prognostic subgroups formed based on these factors [14].

In metastatic melanoma, based on the sites of metastases and serum LDH, patients with metastatic melanoma are classified into eight prognostic groups according to the American Joint Committee on Cancer (AJCC) melanoma TNM classification (currently eighth edition) (M1a-d(0) and M1a-d(1)) [15]. The number of metastatic sites and baseline serum LDH were prognostic for disease progression and mortality in patients with BRAF V600 mutant metastatic melanoma treated with BRAF + MEK inhibitors [7,8,16]. The prognostic performance of primary melanoma features was not reported in these studies. Importantly, primary tumor, Breslow thickness and ulceration are significant prognostic factors in patients with stage I–III melanoma, and there are some publications on the prognostic impact of primary tumor localization and histological subtype in patients with stage IV disease [17,18]. In addition, the prognostic effect of mRNA biomarkers to predict the risk of metastasis improves when combined with clinicopathological data, and some prognostic molecular biomarkers (such as COX-2) correlate with clinicopathological features of melanoma, suggesting that further research is needed [14]. Another marker worth mentioning is serum S100B, which is a routinely monitored laboratory parameter in patients with metastatic melanoma. Several studies have investigated its prognostic effect, but it is not a validated prognostic marker [19]. In our previous study, we found that pT4b primary tumor category, M1c stage or M1d stage at the start of therapy, elevated baseline serum S100B level, and elevated LDH level were associated with poor survival in patients with stage IV melanoma treated with anti-PD-1 [20]. The aim of the present study was to determine the prognostic performance of the above-mentioned factors in patients with unresectable metastatic melanoma treated with BRAF and MEK inhibitors. Since the BRAF + MEK inhibitor combination is what the National Health Insurance Fund reimburses in Hungary for the first-line treatment of BRAF mutant advanced melanoma, 97.5% of the patients included in the study received targeted therapy in the first line.

To determine the independent prognostic effect of clinicopathological factors and serum markers, we used the Cox multivariate proportional-hazards model of disease progression and mortality. Independent determinants of tumor response to BRAF + MEK inhibitor therapy were evaluated using a multivariate logistic regression model. The predictive power of independent prognostic factors for 18 month progression-free survival was evaluated in ROC analysis.

## 2. Materials and Methods

### 2.1. Study Population

In this retrospective study, advanced melanoma patients treated with BRAF and MEK inhibitors at the Department of Dermatology of the University of Debrecen Clinical Center between 2017 and 2024 were selected. The inclusion criteria were the following: BRAF V600 mutation confirmed by a DNA-based diagnostic test, unresectable metastatic melanoma, BRAF + MEK inhibitor combination therapy, and evaluation of tumor response at least once after starting therapy. Adjuvant or metastatic first-line immune checkpoint inhibitor therapy prior to targeted therapy was not an exclusion criterion. Patients who had resectable metastatic disease and received BRAF + MEK inhibitors as an adjuvant treatment after surgery were not included. The source of the data was the integrated hospital information system (MedSolution and UDMED) used at the University of Debrecen (the study was approved by the Regional Ethics Committee; certificate number: 9555-2/2017/EKU).

We recorded the patients’ age and sex, characteristics of the primary tumor (Breslow tumor thickness, ulceration status, localization, Clark invasion level and histological subtype, primary tumor stage according to the eighth edition of the American Joint Committee on Cancer (AJCC) melanoma staging system (pT category) [15], distant metastases according to the eighth edition of the AJCC melanoma staging system (M category), baseline serum S100B and LDH levels, baseline absolute neutrophil and lymphocyte counts in peripheral blood, duration of treatment, and patient death. Imaging test (computer tomography (CT), magnetic resonance imaging (MRI), positron emission tomography with 2-deoxy-2-[fluorine-18]fluoro-D-glucose integrated with computed tomography (18F-FDG PET/CT)) were performed every 3 months. Tumor response to BRAF + MEK inhibitor therapy was evaluated according to the Response Evaluation Criteria in Solid Tumours (RECIST version 1.1) [21]. 

Serum S100B levels were measured using a quantitative automated chemiluminescent immunoassay (LIAISON^®^ S100). The cut-off value, set by the manufacturer, was 0.15 µg/L. An automated colorimetric assay was used to measure serum LDH levels and the cut-off value was the upper limit of normal LDH level as determined by the local laboratory (220 U/L). The absolute neutrophil and lymphocyte counts of the peripheral blood were determined with an automatic hematology analyzer. The baseline neutrophil-to-lymphocyte ratio (NLR) was calculated. Continuous values of NLR were dichotomized using a ROC-optimized cut-off point of 2.33.

### 2.2. Statistical Analysis

Progression-free survival (PFS) and overall survival (OS) were assessed using the Kaplan–Meier Estimator. The PFS (months) was defined as the time from the start of therapy to disease progression or to the last moment of follow-up. OS (months) was calculated by taking into account the time from the start of therapy to the last time of follow-up or death. The comparison of survival probabilities was performed using a two-sided Log-rank test. Median survival time (months) is presented with 95% confidence interval (95% CI). Univariate and multivariate hazard ratios (HR) were calculated with the corresponding upper and lower 95% confidence intervals using Cox regression analysis [22]. Furthermore, prognostic factors related to tumor response were evaluated using multivariate logistic regression models. Missing values were removed from the dataset used for statistical analyses. Sources of missing data were: no evidence of primary tumor, no full histopathology report available, and missing baseline laboratory parameters. The odds ratio (OR) was calculated with corresponding upper and lower 95% confidence intervals. Receiver operating characteristic (ROC) curve analysis was performed to investigate the discriminative power of combinations of prognostic factors on disease progression [23]. The area under the curve (AUC) derived from the ROC curve was shown with 95% confidence intervals (95% CI). The interpretation of the AUC is the following: an AUC of 0.5 indicates no discrimination, 0.7 to 0.8 is considered as acceptable, 0.8 to 0.9 is considered as excellent, and greater than 0.9 is considered as outstanding. To select the optimal cut-off point for NLR, we used the ROC curve for baseline NLR for predicting disease progression. The Youden index derived from the ROC curve was chosen as the NLR threshold.

The significance level was *p* < 0.05 in all cases. All statistical analyses were performed using IBM SPSS Statistics for Windows, version 25.0 (IBM Corp., Armonk, NY, USA) by a biostatistician, E.A. Janka.

## 3. Results

### 3.1. Patient and Disease Characteristics

A total of 199 patients with unresectable stage IV melanoma treated with BRAF and MEK inhibitors (58.8% male) were included in the survival analysis. A total of 88.9% of the patients (N = 177) received a combination of dabrafenib + trametinib and 11.1% (N = 22) received a combination of vemurafenib + cobimetinib. The majority of patients (N = 194) received the treatment in the first line. The characteristics of the patients and their disease are summarized in Table 1. The average age of the patients at the start of therapy was 62.98 ± 13.31 years. The Eastern Cooperative Oncology Group (ECOG) performance status of the patients was 0 or 1. The Breslow tumor thickness of the primary tumor was available in 81.9% of patients (N = 163). The mean tumor thickness was 5.43 ± 5.32 mm (median [inter-quartile range (IQR)]: 4.00 [2.20; 7.00]). 

At the start of therapy, 32.1% of patients had stage M1a disease, 18.1% had stage M1b disease, 35.2% of individuals had stage M1c disease, and 14.6% of patients had stage M1d disease. Elevated baseline serum LDH and S100B levels were observed in 63.3% and 42.7% of patients, respectively. Baseline NLR was above the cut-off in 53.8% of patients.

In our study population, 57.8% (37/64) of patients with stage M1a, 66.7% (24/36) of patients with stage M1b, 82.9% (58/70) of patients with stage M1c, and 86.2% (25/29) of patients with stage M1d experienced progression during the observation period. The median PFS was 7.39 months [(IQR): 3.14–17.54]. A total of 61 patients received subsequent anti-PD-1 ± anti-CTLA-4 therapy after progression on targeted treatment. The median OS for the entire study population was 15.14 months [(IQR): 7.82–31.64]. For patients who were alive at the end of the observation period (N = 83), the median follow-up time was 23.86 months ((IQR): 11.21–50.86) from the start of treatment.

### 3.2. M Stage, Serum S100B, pT Category, Clark Level and Primary Tumor Localization Are Significant and Independent Prognostic Factors of Disease Progression in Metastatic Melanoma Patients Treated with BRAF + MEK Inhibitors

In our study, the PFS was not significantly different when comparing male and female patients, nor was it significantly different when comparing patients under 60 years of age and patients over 60 years of age (Figures S1A and S2A).

**Figure 1:**
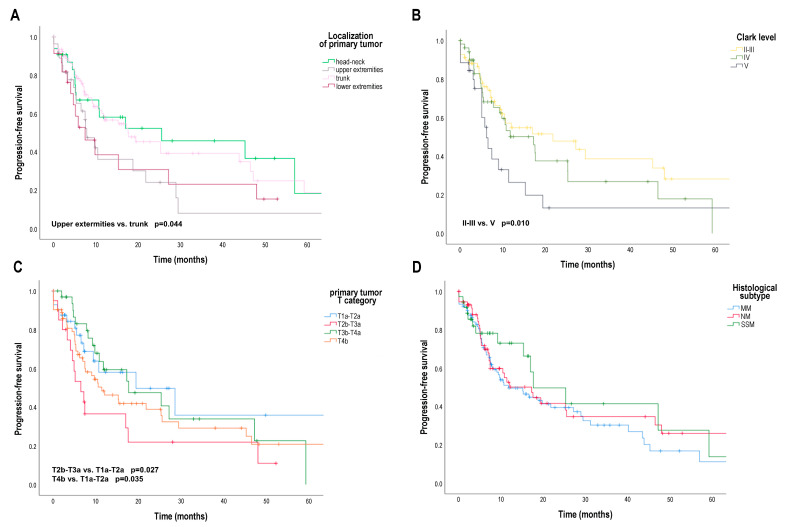
Progression-free survival (PFS) in patients with metastatic melanoma treated with BRAF and MEK inhibitors according to primary tumor localization, Clark invasion level, AJCC 8th edition primary tumor (pT) category and histological subtype. (**A**) PFS (months) according to primary tumor localization (head-neck, upper extremities, lower extremities, trunk); (**B**) PFS (months) according to Clark invasion level (II–III, IV, V); (**C**) PFS (months) according to pT category (T1a–T2a, T2b–T3a, T3b–T4a, T4b); (**D**) PFS (months) according to primary tumor histological subtype (MM, SSM, NM). AJCC—American Joint Committee on Cancer, MM—unclassified malignant melanoma or no evidence of primary tumor, SSM—superficial spreading melanoma; NM—nodular melanoma. Survival probabilities were compared using a two-sided log-rank test.

Interestingly, regarding the characteristics of the primary tumor, we found significant differences for PFS (Figure 1). The median PFS was 25.50 months [95% CI 2.00; 40.09] for head and neck localization of the primary tumor, 17.64 months [95% CI 5.94; 29.35] for trunk localization, but only 7.79 months [95% CI 1.99; 12.80] for lower extremity localization and 7.39 months [95% CI 4.71; 10.86] for upper extremity localization. Comparing the PFS of patients for primary melanoma with upper extremity localization and primary melanoma with trunk localization, the difference was significant (*p* = 0.044) (Figure 1A). The median PFS was significantly worse (*p* = 0.010) for Clark invasion level V (6.14 months [95% CI 4.67; 7.61]) than for Clark invasion level II–III (21.75 months [95% CI 3.65; 39.85]). The median PFS of patients with Clark level IV primary tumors was 17.21 months [95% CI 9.17; 25.26] (Figure 1B). The AJCC eighth edition pT category was also found to be a factor affecting PFS. Median PFS was significantly worse for pT2b–T3a primary melanoma (6.43 months [95% CI 3.22; 9.64]) than for pT1a–T2a primary melanoma (19.39 months [95% CI 10.59; 26.58]) (*p* = 0.027) and significantly worse for pT4b primary melanoma (11.36 months [95% CI 4.71; 18.00]) than for pT1a–T2a primary melanoma (*p* = 0.035) (Figure 1C). The histological subtype of the primary tumor had no effect on PFS (Figure 1D). The risk of progression was significantly increased by the limb localization of the primary tumor compared to trunk localization (upper extremity: HR 3.70 [95% CI 1.81; 7.56]; lower extremity: HR 1.19 [95% CI 1.03; 2.51]), Clark invasion level V compared to Clark Invasion level II–III (HR 3.32 [95% CI 1.33; 8.30]), pT2b–T3a and pT4b categories compared to pT1a–T2a category (pT2b–T3a: HR 2.43 [95% CI 1.51; 7.77]; pT4b: HR 1.84 [95% CI 1.01; 4.38]) (Table 2).

**Figure 2:**
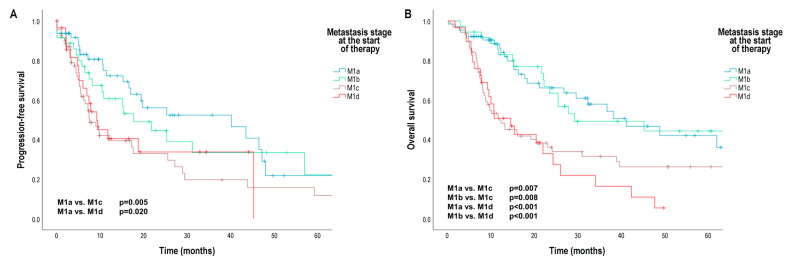
Progression-free survival (PFS) and overall survival (OS) in patients with metastatic melanoma treated with BRAF and MEK inhibitors according to AJCC 8th edition distant metastasis (M) stage. (**A**) PFS (months) according to M stage; (**B**) OS (months) according to M stage. AJCC—American Joint Committee on Cancer. Survival probabilities were compared using a two-sided log-rank test.

Median PFS was significantly worse in patients with non-central nervous system visceral metastases (M1c) (7.50 months [95% CI 4.57; 10.44]) and central nervous system metastases with or without any other distant sites of disease (M1d) (9.18 months [95% CI 4.36; 13.99]) than in patients with metastases in the skin, subcutaneous tissue, or distant lymph nodes (M1a) (40.14 months [95% CI 11.81; 68.48]) (M1a vs. M1c: *p* = 0.005; M1a vs. M1d: *p* = 0.020) (Figure 2A). The risk of progression was significantly increased in patients with M1c and M1d stage compared to M1a stage (M1c: HR 3.09 [95% CI 1.58; 6.04]; M1d: HR 4.07 [95% CI 1.80; 9.18]) (Table 2). 

**Figure 3:**
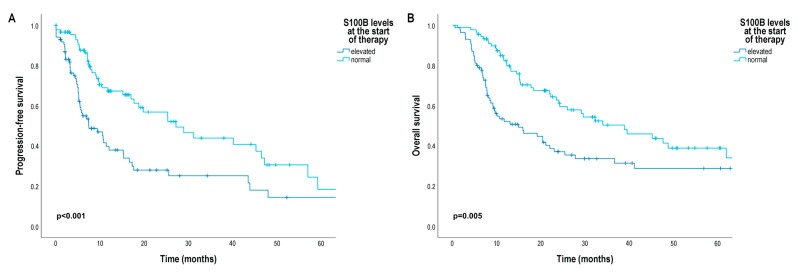
Progression-free survival (PFS) and overall survival (OS) in patients with metastatic melanoma treated with BRAF and MEK inhibitors according to baseline serum S100B level. (**A**) PFS (months) according to baseline serum S100B level; (**B**) OS (months) according to baseline serum S100B level. Survival probabilities were compared using a two-sided log-rank test.

Patients with elevated baseline serum S100B levels had a significantly shorter median progression-free survival (7.43 months [95% CI3.07; 11.79]) than patients with normal baseline serum S100B levels (27.14 months [95% CI 15.33; 38.95]) (*p* < 0.001) (Figure 3A). The risk of progression was significantly increased in patients with elevated baseline serum S100B level compared to normal baseline serum S100B level (HR 1.83 [95% CI 1.09; 3.63]) (Table 2). 

**Figure 4:**
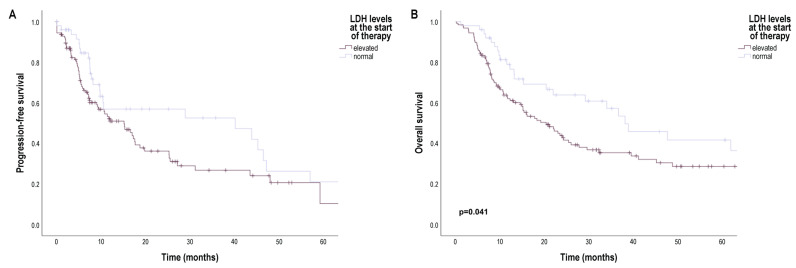
Progression-free survival (PFS) and overall survival (OS) in patients with metastatic melanoma treated with BRAF and MEK inhibitors according to baseline serum LDH level. (**A**) PFS (months) according to baseline serum LDH level; (**B**) OS (months) according to baseline serum LDH level. LDH—lactate dehydrogenase. Survival probabilities were compared using a two-sided log-rank test.

Patients with elevated baseline serum LDH levels also had a worse median PFS (15.14 months [95% CI 9.86; 20.43]) than patients with normal baseline serum LDH levels (40.14 months [95% CI 20.63; 80.58]), but the difference was not significant (*p* = 0.059) (Figure 4A). In addition, median PFS was worse in patients with a baseline NLR above the cut-off point (10.50 months [95% CI 5.24; 15.76]) than in patients with a baseline NLR below the cut-off point (25.50 months [95% CI 9.25; 31.75]), but the difference was not significant (*p* = 0.070) (Figure S3A).

### 3.3. M Stage, pT Category and Clark Level Are Significant and Independent Determinants of Tumor Response to BRAF + MEK Inhibitor Treatment in Patients with Metastatic Melanoma

Patients with stage M1a had a higher chance of achieving CR-PR status than patients with stage M1d (CR-PR vs. SD: OR 7.67 [95% CI 1.10; 18.86] M1a vs. M1d; CR-PR vs. PD: OR 8.48 [95% CI 1.21; 15.78] M1a vs. M1d) (Table 3). Furthermore, achieving CR-PR status was more likely in metastatic disease from pT1a–T2a primary tumor than in metastatic disease from pT2b–T3a or pT4b primary tumor (CR-PR vs. PD: OR 4.71 [95% CI 1.13; 10.43] T1a–T2a vs. T2b–T3a; CR-PR vs. PD: OR 3.84 [95% CI 1.12; 11.54] T1a–T2a vs. T4b). Achieving CR-PR status was more likely in metastatic disease from Clark level II–III primary melanoma than in metastatic disease from Clark level V melanoma (CR-PR vs. PD: OR 7.46 [95% CI 1.17; 12.30] Clark level II–III vs. Clark level V).

### 3.4. Diagnostic Impact of Independent Prognostic Factors of Disease Progression on the Prediction of 18 Month Progression-Free Survival

**Figure 5:**
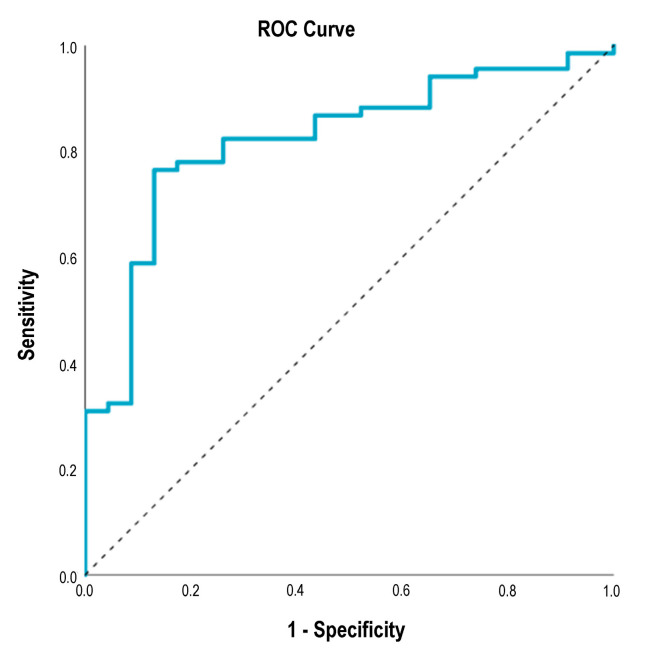
ROC curve for the combination of primary tumor localization, Clark invasion level, pT category, baseline M stage, and baseline serum S100B level discriminating BRAF- and MEK-inhibitor-treated metastatic melanoma patients with progression-free survival of less than 18 months versus more than 18 months. AUC 0.822 [95% CI 0.727; 0.916], *p* < 0.001. ROC—receiver operating characteristic, AUC—area under the curve.

Patients who were followed for at least 18 months and patients who experienced disease progression during the study period were included in ROC analysis to assess the discriminative power of the combination of independent prognostic factors identified in Cox regression for disease progression. The combination of primary tumor localization, Clark invasion level, pT category, baseline M stage, and baseline serum S100B level resulted in an excellent AUC value of 0.822 [95% CI 0.727; 0.916], *p* < 0.001 (Figure 5). This means that the combined assessment of negative prognostic factors adequately differentiates patients with a progression-free survival of less than 18 months from patients with a progression-free survival of more than 18 months. The analysis was also performed for 6 month and 12 month PFS. The diagnostic effect of the combination of prognostic factors remained; however, the AUC values were somewhat lower (6 month PFS: AUC 0.711 [0.592; 0.831]; *p* = 0.002; 12 month PFS: AUC 0.728 [95% CI 0.566; 0.998]; *p* = 0.024).

### 3.5. M Stage, Serum S100B, Serum LDH, Clark Level and Primary Tumor Localization Are Significant and Independent Prognostic Factors of Mortality in Metastatic Melanoma Patients Treated with BRAF + MEK Inhibitors

We did not find significant differences for OS by sex or by age group (Figures S1B and S2B).

In Kaplan–Meier analysis, we did not found significant differences in patients’ OS according to the primary tumor characteristics (Figure S4), but as expected, median OS was significantly shorter in patients with stage M1c (12.25 months [95% CI 6.39; 18.11]) and M1d (14.50 months [95% CI 8.02; 20.98]) than in patients with stage M1a (41.14 months [95% CI 23.99; 58.29]) (M1a vs. M1c: *p* = 0.007; M1a vs. M1d: *p* = 0.008) and M1b (metastases in the skin, subcutaneous tissue, or distant lymph nodes and/or lung metastases) (29.18 months [95% CI 14.43; 47.43]) (M1b vs. M1c: *p* < 0.001; M1b vs. M1d: *p* < 0.001) (Figure 2B). Furthermore, median OS was also significantly worse in patients with elevated baseline serum S100B levels (14.93 months [95% CI 5.71; 24.15]) than in patients with normal baseline serum S100B levels (38.89 months [95% CI 22.94; 54.84]) (*p* = 0.005) (Figure 3B), and median OS was significantly shorter in patients with elevated baseline serum LDH levels (20.35 months [95% CI 13.51; 27.20]) than in patients with normal baseline serum LDH levels (38.11 months [95% CI 23.49; 52.72]) (*p* = 0.041) (Figure S4). Median OS was significantly worse in patients with a baseline NLR above the cut-off point (20.36 months [95% CI 12.28; 28.44]) than in patients with a baseline NLR below the cut-off point (38.11 months [95% CI 15.29; 60.92]) (*p* = 0.024) (Figure S3B).

Limb localization of the primary tumor compared to trunk localization (upper extremity: HR 3.20 [95% CI 1.55; 6.18]; lower extremity: HR 1.64 [95% CI 1.06; 4.03]), Clark invasion level V compared to Clark Invasion level II–III (HR 4.23 [95% CI 1.72; 8.40]), M1c and M1d stage compared to M1a stage (M1c: HR 2.47 [95% CI 1.24; 4.90]; M1d: HR 4.16 [95% CI 1.95; 8.88]), elevated baseline serum S100B level compared to normal baseline serum S100B level (HR 2.01 [95% CI 1.18; 3.43]), and elevated baseline serum LDH level compared to normal baseline serum LDH level (HR 1.84 [95% CI 1.08; 2.32]) were associated with a higher risk of death (Table 4).

## 4. Discussion

The pathogenesis of melanoma is complex, which means that cutaneous melanomas with different biological behavior can develop as a result of the interaction of environmental factors, genetic susceptibility and other host factors [3,24,25,26]. The latest clinicopathological classification of melanomas differentiates between melanoma subtypes based on the role of UV radiation, clinical and morphological features, data on the genetic evolution of melanomas from precursor lesions, and molecular pathological classification data [27]. The most common gene mutations in cutaneous melanoma affect the activity of the mitogen-activated protein kinase (MAPK) signaling pathway, which is one of the main regulators of cell proliferation, differentiation and survival [5,28,29]. BRAF mutation, which can be detected in 35–50% of melanomas, is a frequent oncogenic event in melanomas that develop on sun-exposed skin without pronounced solar elastosis, regardless of histological subtypes [27,30]. V600E mutation occurs in 74–86% of cases, V600K in 10–30%, and V600M/D/R in 3–5% [31]. Interindividual heterogeneity in the clinicopathological features of BRAF mutated melanoma is associated with additional genetic alterations, such as TERT promoter mutations, biallelic inactivation of CDKN2A, loss of PTEN, TP53 mutations, copy number changes, increased genomic instability, and epigenetic events and factors derived from the tumor stroma [31,32,33,34]. In the background of BRAF inhibitor resistance, the reactivation of the MAPK pathway can most often be detected, in addition to the activation of parallel signaling pathways and epigenetic changes [5,31,32,33,35,36]. Furthermore, intratumoral and intertumoral heterogeneity can be mentioned as a contributing factor to the development of resistance to BRAF inhibitor therapy in a metastatic patient [37]. Treatment with a combination of a BRAF inhibitor and a MEK inhibitor is a strategy that moderates the effects of tumor heterogeneity and thus results in better PFS of patients compared to BRAF inhibitor monotherapy [38]. Interestingly, a pilot study found that in BRAF + MEK inhibitor-resistant melanoma, chemotherapy can be a successful treatment modality if it induces pyroptosis, an immunogenic form of cell death [39]. This confirms that tumor regression in metastatic melanoma is highly dependent on the antitumor immune response developed during treatment, and this does not only apply to immunotherapies [13,39]. High expression of genes related to the immune response was more frequent in melanomas of patients who achieved complete remission with BRAF and MEK inhibitors, while high expression of keratin and kallikrein genes was more frequent in melanomas of patients with rapid progression [13]. Inhibition of relevant immunosuppressive pathways identified in the tumor microenvironment (e.g., PD-1, indoleamine-2,3-dioxygenase 1 (IDO1)) can be an effective strategy to strengthen antitumor immunity and stop tumor growth [40,41]. Of note, experimental data suggest that BRAF inhibitor treatment can reduce IDO1 expression [39,42], while high IDO1 levels can be detected in melanoma samples in connection with the development of resistance to the BRAF inhibitor [42]. A deeper insight into the post-translational regulatory mechanisms affecting the activity and half-life of IDO1 may contribute to improving therapeutic efficacy [41,43]. In this single-center retrospective study, we analyzed the prognostic performance of clinicopathological features and routinely available blood biomarkers in patients with BRAF V600 mutant unresectable advanced melanoma treated with BRAF + MEK inhibitor.

Data from clinical trials showed that female sex and older age were associated with better PFS and OS in patients with metastatic melanoma treated with dabrafenib + trametinib than male sex and younger age [7]. However, we found no significant difference in patient survival when comparing male and female patients, or between patients under 60 years and over 60 years of age.

As a new result, we found that among the clinicopathological characteristics of the primary tumor, the Clark level of invasion and the localization of melanoma are significant and independent prognostic factors for both PFS and OS in metastatic patients treated with BRAF and MEK inhibitors. The AJCC eighth edition pT category was an independent determinant of PFS. Furthermore, Clark invasion level and pT category had an independent significant effect on tumor response to BRAF + MEK inhibitor treatment. We previously found that pT category was an independent prognostic factor for survival in patients with metastatic melanoma treated with anti-PD-1 [20]. It is known that the characteristics of the primary tumor influence disease-free and overall survival in stages I–II, but we can only guess how their independent prognostic value remains in metastatic melanoma [44,45]. Of note, pT category is known to affect the prognosis of patients with lymph node metastases [45]. A possible explanation for the observed association between primary melanoma characteristics and patient outcome is that high-risk pT categories, Clark levels, mitotic counts, and regional lymph node metastases correlate with BRAF V600E nuclear translocation [46]. Nuclear localization of BRAF V600E in melanoma cells has been shown to be associated with aggressive biological behavior and BRAF inhibitor resistance [46]. Further analyzes revealed overexpression of heme oxygenase 1 and the activation of the AKT pathway in association with nuclear BRAF V600E and cell proliferation in BRAF inhibitor resistant melanoma cells [47]. Interestingly, in our study, limb localization of primary melanoma was associated with a higher risk of progression and death than trunk localization of the primary tumor. In another study, half of patients with extremity melanoma did not respond at all to immune checkpoint inhibitor therapy, and primary tumor localization was an independent and significant prognostic factor for OS [17]. It can be assumed that region-specific differences in endogenous and exogenous factors affecting the skin may contribute to differences in the biological behavior of primary tumors localized in different skin regions [24,48].

In a previous study, the nodular histological subtype of primary melanoma was found to be an independent and significant prognostic factor for mortality in metastatic patients treated with BRAF ± MEK inhibitors [18]. Although in our study we did not find a statistically significant difference in patient survival according to the histological subtypes, the results of the aforementioned study support our findings that the clinicopathological characteristics of the primary tumor can be of prognostic value even in the case of metastatic disease.

In patients with metastatic melanoma, the site of distant metastases, tumor volume, and serum LDH are well-known prognostic factors for mortality [15,49,50]. In our present study, we found that PFS and OS were significantly better in patients with stage M1a than in patients with stage M1c or M1d when treated with BRAF + MEK inhibitors. In addition, patients with normal baseline serum LDH had a better prognosis than patients with elevated baseline serum LDH; however, baseline serum LDH was found to be a significant and independent prognostic factor for OS but not for PFS. These results are consistent with literature data. In a study evaluating patients with metastatic melanoma treated with dabrafenib, baseline tumor volume was found to be a prognostic factor for PFS and OS in multivariate analysis [51]. Three or more metastatic sites and brain metastases have been reported to be associated with shorter PFS in patients treated with dabrafenib + trametinib [16,52]. Normal baseline serum LDH and <3 metastatic sites were associated with significant PFS and OS benefit in patients with unresectable advanced melanoma treated with BRAF + MEK inhibitors [7,8]. Metastatic pattern and pretreatment LDH level have also been shown to be independent prognostic factors in other retrospective studies analyzing survival data in BRAF V600 mutant metastatic melanoma patients treated with first-line immune checkpoint inhibitors or BRAF + MEK inhibitors [53,54]. Furthermore, in a study evaluating the prognostic significance of serum LDH as a function of BRAF mutation status, it was shown that elevated serum LDH levels are associated with poor outcomes in patients with metastatic melanoma in both BRAF mutant and BRAF wild-type cases [55]. Overall, the site of metastasis and serum LDH level appear to be strong determinants of melanoma prognosis, independent of BRAF mutational status and type of treatment. A correlation between clinical outcome and antitumor immunity has been demonstrated in both BRAF + MEK inhibitor-treated and anti-PD-1-treated metastatic melanoma patients [13,39,56]. One possible explanation for our results is that the characteristics of metastatic disease may reflect a greater or lesser chance of a therapy-induced antitumor immune response [57].

Serum S100B levels correlate with aggressive disease and have been shown to be a reliable prognostic marker in metastatic melanoma [19,20]. In addition, distant metastases were significantly more likely to be associated with elevated serum S100B levels in patients with BRAF mutant melanoma than in patients with BRAF wild-type melanoma [55]. However, in contrast to metastatic patients with BRAF wild-type melanoma, serum S100B was not a prognostic factor for OS in metastatic patients with BRAF mutant melanoma [55]. In our study, we found that elevated baseline serum S100B levels were associated with a higher risk of progression and death compared with normal baseline serum S100B levels in BRAF V600 mutant metastatic melanoma patients treated with BRAF + MEK inhibitors. This result is consistent with a previous report in which serum S100B was found to be a prognostic factor for disease-specific survival in BRAF inhibitor-treated metastatic melanoma patients [58]. In addition, measurement of serum S100B levels has been shown to be useful in monitoring tumor response in patients receiving targeted therapy [58,59]. According to literature data, the neutrophil-to-lymphocyte ratio can be an independent prognostic factor in metastatic melanoma treated with BRAF + MEK inhibitor [60,61,62]. In the melanoma cohort that we analyzed, baseline NLR proved to be a significant prognostic factor for OS in univariate analysis, but its prognostic power was lost in multivariate analysis. Similarly, in another study, baseline NLR was a prognostic factor for PFS in patients with BRAF mutant metastatic melanoma, regardless of the therapy received, but only in univariate analysis [63]. The chance of long-term effectiveness of a particular therapy is very important. Therefore, we examined the predictive power of independent prognostic factors of disease progression for 18 month PFS in ROC analysis. The combination of primary tumor localization, Clark invasion level, pT category, baseline M stage, and baseline serum S100B level was excellent at distinguishing patients who were likely to be progression-free beyond 18 months from those who were not. This is a promising result, but validation is needed. 

The analysis of prognostic factors related to disease progression is of clinical importance because it contributes to a better understanding of disease biology and helps to optimize treatment decisions in the long term. The strength of our study is that it used logistic regression models, and with the analysis we were able to identify independent prognostic factors that have not yet been described in relation to metastatic melanoma treated with BRAF + MEK inhibitors, which are worthy of further investigation. The limitation of our study is that it was a retrospective study, and the proposed hypotheses must be verified in prospective studies. Although metastatic melanoma patients treated with BRAF + MEK inhibitors at our center were included in the study without selection, confounding variables may remain hidden due to the single-center study design. For example, socioeconomic factors and ambient UV radiation, which can influence both exposures and health outcomes, may vary by geographic region [64]. Further studies with larger numbers of cases and independent melanoma cohorts are needed. The number of patients was not high enough and the follow-up time was not long enough to analyze the data of the subgroup of patients who received immunotherapy after targeted therapy.

## 5. Conclusions

Stratification of patients according to prognostic biomarkers may increase the success of therapy [14]. Our study suggests that clinicopathological factors, including primary tumor characteristics, stage of metastatic disease, and serum LDH and S100B, can be used in the prognostic stratification of metastatic melanoma patients treated with BRAF and MEK inhibitors. These results need to be validated, but it can be emphasized that in daily clinical practice it is worth paying attention to all clinicopathological parameters. We believe that combining these prognostic factors with molecular biomarkers (e.g., circulating tumor DNA [65]) in a multivariate prognostic model should be considered [34]. Furthermore, we found that BRAF V600 mutant primary melanoma that localizes to the extremities or invades the subcutaneous tissue may represent distinct melanoma subtypes that merit further investigation.

## Figures and Tables

**Table 1:** Characteristics of metastatic melanoma patients treated with BRAF and MEK inhibitors.

Patient Characteristics	N (%)
Total	199 (100.0)
Age distribution	
<60 years	79 (39.7)
≥60 years	120 (60.3)
Sex	
Male	117 (58.8)
Female	82 (41.2)
**Primary Melanoma Characteristics**	**N (%)**
Localization	
Head and neck	22 (11.1)
Upper limbs	27 (13.6)
Lower limbs	33 (16.6)
Trunk	93 (46.6)
Occult	24 (12.1)
Histological subtype	
SSM	36 (18.1)
LMM	1 (0.5)
NM	73 (36.7)
MM	89 (44.7)
Clark level	
II	3 (1.5)
III	51 (25.6)
IV	69 (34.7)
V	26 (13.0)
unknown	50 (25.2)
AJCC 8th edition T category	
pT1a	8 (4.0)
pT1b–T2a	25 (12.6)
pT2b–T3a	20 (10.0)
pT3b–T4a	42 (21.1)
pT4b	72 (36.2)
unknown	32 (16.1)
**Disease Characteristics**	
AJCC 8th edition M category at the start of treatment	
M1a	64 (32.1)
M1b	36 (18.1)
M1c	70 (35.2)
M1d	29 (14.6)
Baseline serum LDH level	
normal	50 (25.1)
elevated	126 (63.3)
unknown	23 (11.6)
Baseline serum S100B level	
normal	90 (45.2)
elevated	85 (42.7)
unknown	24 (12.1)
Baseline peripheral blood NLR	
below cut-off	62 (31.2)
above cut-off	107 (53.8)
unknown	30 (15.0)

N—number of cases; SSM—superficial spreading melanoma; LMM—lentigo maligna melanoma; NM—nodular melanoma; MM—unclassified malignant melanoma or no evidence of primary tumor; AJCC—American Joint Committee on Cancer; T—primary tumor; M—distant metastasis, NLR—neutrophil-to-lymphocyte ratio.

**Table 2:** Independent determinants of disease progression in patients with metastatic melanoma treated with BRAF and MEK inhibitors by Cox proportional-hazards model.

Cox Regression According to Endpoint: PFS		Univariate Logistic Regression Model		Multivariate Logistic Regression Model	
Variables	Categories	HR [95% CI]	*p*-Value	HR [95% CI]	*p*-Value
Age	≥60 years/<60 years	1.04 [0.72; 1.49]	0.842	-	-
Sex	male/female	1.21 [0.83; 1.76]	0.317	-	-
Histological subtype	SSM/MM	0.99 [0.60; 1.62]	0.996	-	-
	NM/MM	1.07 [0.58; 1.30]	0.865		
Localization of primary tumor	head and neck/trunk	0.84 [0.48; 1.47]	0.530	0.84 [0.40; 1.75]	0.637
	upper extremities/trunk	**1.73 [1.05; 2.86]**	**0.032**	**3.70 [1.81; 7.56]**	**<0.001**
	lower extremities/trunk	**1.57 [1.01; 2.77]**	**0.049**	**1.19 [1.03; 2.51]**	**0.044**
Clark level	IV/II–III	1.02 [0.46; 1.19]	0.212	1.01 [0.66; 1.28]	0.219
	V/II–III	**1.70 [1.02; 2.98]**	**0.048**	**3.32 [1.33; 8.30]**	**0.010**
AJCC 8th edition T category	pT2b–T3a/pT1a–T2a	**1.77 [1.02; 3.39]**	**0.045**	**2.43 [1.51; 7.77]**	**0.003**
	pT3b–T4a/pT1a–T2a	1.03 [0.55; 1.92]	0.923	1.14 [0.51; 2.55]	0.744
	pT4b/pT1a–T2a	**1.15 [1.01; 1.93]**	**0.048**	**1.84 [1.01; 4.38]**	**0.049**
AJCC 8th edition M category at the beginning of therapy	M1b/M1a	1.17 [0.67; 2.04]	0.586	1.34 [0.56; 3.23]	0.516
	M1c/M1a	**2.01 [1.26; 3.21]**	**0.003**	**3.09 [1.58; 6.04]**	**0.001**
	M1d/M1a	**2.02 [1.15; 3.55]**	**0.014**	**4.07 [1.80; 9.18]**	**0.001**
Serum S100B level	elevated/normal	**1.83 [1.25; 2.68]**	**0.002**	**1.83 [1.09; 3.63]**	**0.032**
Serum LDH level	elevated/normal	1.47 [0.96; 2.26]	0.078	-	-
Peripheral blood NLR	above/below cut-off	1.49 [0.96; 2.31]	0.072		

Significant results are in bold. HR [95% CI]—hazard ratio with 95% confidence intervals; SSM—superficial spreading melanoma; NM—nodular melanoma; MM—unclassified malignant melanoma or no evidence of primary tumor; AJCC—American Joint Committee on Cancer; T—primary tumor; M—distant metastasis, NLR—neutrophil-to-lymphocyte ratio.

**Table 3:** Independent determinants of tumor response to BRAF + MEK inhibitor treatment in patients with metastatic melanoma by multivariate logistic regression model.

Multivariate Logistic Regression Model				
	Variables	Categories	OR [95% CI]	*p*-Value
CR-PR vs. SD	AJCC 8th edition M category at the beginning of therapy	M1a/M1d	7.67 [1.10; 18.86]	0.045
CR-PR vs. PD	AJCC 8th edition T category	T1a–T2a/T2b–T3a	4.71 [1.13; 10.43]	0.045
		T1a–T2a/T4b	3.84 [1.12; 11.54]	0.049
	Clark level	II–III/V	7.46 [1.17; 12.30]	0.036
	AJCC 8th edition M category at the beginning of therapy	M1a/M1d	8.48 [1.21; 15.78]	0.035

The adjustment factors used in the multivariate model were those that were found to be significant in the univariate logistic regression model. Only the significant results and categories are shown in this table. CR-PR—complete/partial remission, SD—stable disease, PD—progressive disease, OR [95% CI]—odds ratio with 95% confidence intervals; AJCC—American Joint Committee on Cancer; T—primary tumor; M—distant metastasis.

**Table 4:** Independent determinants of mortality in patients with metastatic melanoma treated with BRAF and MEK inhibitors by Cox proportional-hazards model.

Cox Regression According to Endpoint: OS		Univariate Logistic Regression Model		Multivariate Logistic Regression Model	
Variables	Categories	HR [95% CI]	*p*-Value	HR [95% CI]	*p*-Value
Age	≥60 years/<60 years	1.20 [0.83; 1.75]	0.339	-	-
Sex	male/female	1.44 [0.98; 2.11]	0.060	-	-
Histological subtype	SSM/MM	0.99 [0.65; 1.50]	0.955	-	-
	NM/MM	1.02 [0.62; 1.68]	0.946		
Localization of primary tumor	head and neck/trunk	0.97 [0.56; 1.82]	0.975	0.86 [0.41; 1.78]	0.677
	upper extremities/trunk	**1.24 [1.03; 2.09]**	**0.001**	**3.20 [1.55; 6.18]**	**0.004**
	lower extremities/trunk	**1.13 [1.01; 2.56]**	**0.045**	**1.64 [1.06; 4.03]**	**0.040**
Clark level	IV/II–III	1.15 [0.72; 1.85]	0.566	1.69 [0.91; 3.14]	0.099
	V/II–III	**1.73 [1.05; 3.13]**	**0.048**	**4.23 [1.72; 8.40]**	**0.002**
AJCC 8th edition T category	pT2b–T3a/pT1a–T2a	**1.20 [1.01; 2.37]**	**0.038**	1.15 [0.82; 2.77]	0.067
	pT3b–T4a/pT1a–T2a	1.06 [0.58; 1.95]	0.846	1.05 [0.47; 2.40]	0.913
	pT4b/pT1a–T2a	**1.14 [1.02; 1.77]**	**0.024**	1.22 [0.84; 2.76]	0.062
AJCC 8th edition M category at the beginning of therapy	M1b/M1a	0.92 [0.51; 1.67]	0.794	1.25 [0.53; 2.94]	0.604
	M1c/M1a	**1.94 [1.21; 3.11]**	**0.006**	**2.47 [1.24; 4.90]**	**0.010**
	M1d/M1a	**2.61 [1.50; 4.55]**	**0.001**	**4.16 [1.95; 8.88]**	**<0.001**
Serum S100B level	elevated/normal	**1.78 [1.19; 2.65]**	**0.005**	**2.01 [1.18; 3.43]**	**0.010**
Serum LDH level	elevated/normal	**1.65 [1.04; 2.61]**	**0.033**	**1.84 [1.08; 2.32]**	**0.025**
Peripheral blood NLR	above/below cut-off	**1.63 [1.06; 2.51]**	**0.025**	1.32 [0.77; 2.27]	0.311

Significant results are in bold. HR [95% CI]—hazard ratio with 95% confidence intervals; SSM—superficial spreading melanoma; NM—nodular melanoma; MM—unclassified malignant melanoma or no evidence of primary tumor; AJCC—American Joint Committee on Cancer; T—primary tumor; M—distant metastasis, NLR—neutrophil-to-lymphocyte ratio.

## Data Availability

The original contributions presented in the study are included in the article/Supplementary Material, further inquiries can be directed to the corresponding author.

## Supplementary Materials

The following supporting information can be downloaded at: https://www.mdpi.com/article/10.3390/cancers16172981/s1, Figure S1: Progression-free survival (PFS) and overall survival (OS) in patients with metastatic melanoma treated with BRAF and MEK inhibitors by sex; Figure S2: Progression-free survival (PFS) and overall survival (OS) in patients with metastatic melanoma treated with BRAF and MEK inhibitors by age group; Figure S3: Progression-free survival (PFS) and overall survival (OS) in patients with metastatic melanoma treated with BRAF and MEK inhibitors according to baseline peripheral blood NLR; Figure S4: Overall survival (OS) in patients with metastatic melanoma treated with BRAF and MEK inhibitors according to primary tumor localization, Clark invasion level, AJCC 8th edition primary tumor (pT) category and histological subtype.
