# Supplementary material for: Prognostic Role of Clinicopathological Characteristics and Serum Markers in Metastatic Melanoma Patients Treated with BRAF and MEK Inhibitors
Source: Cancers (Basel). 2024 Aug 27;16(17):2981. doi: 10.3390/cancers16172981 (PMC11393897; doi:10.3390/cancers16172981)
Supplement: Supplementary file 1 [file cancers-16-02981-s001.zip › cancers-3161416-supplementary.pdf]

Supplementary Materials

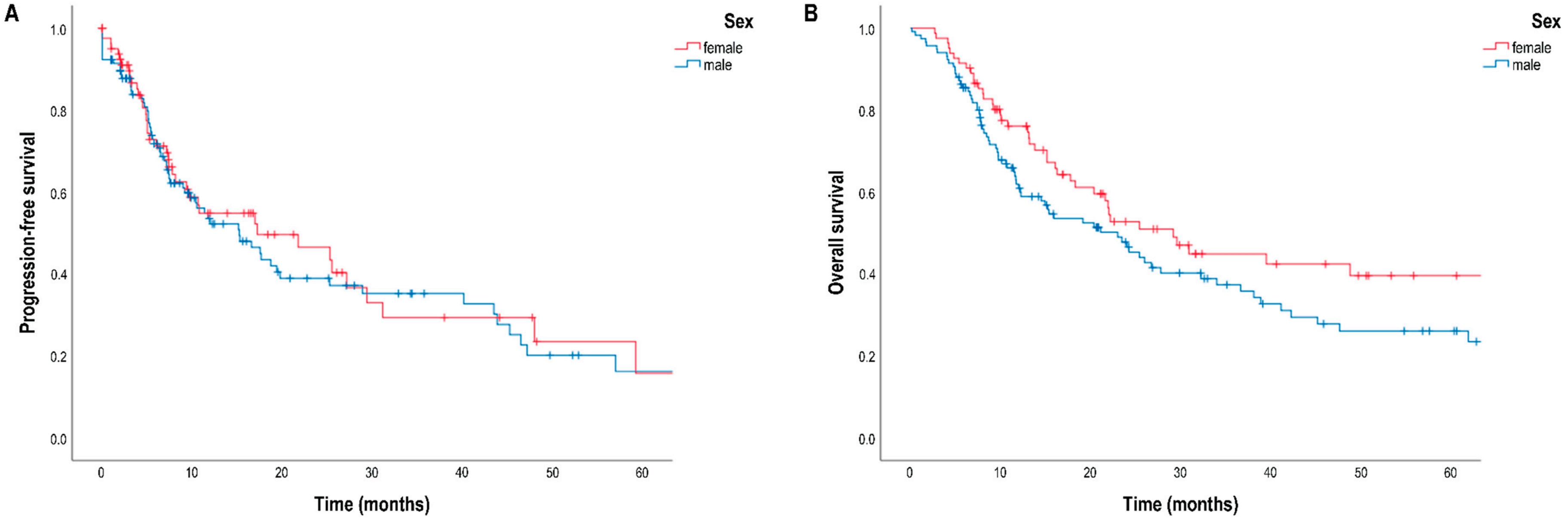

Supplementary Figure S1. Progression-free survival (PFS) and overall survival (OS) in patients with metastatic melanoma treated with BRAF and MEK inhibitors by sex. (**A**) PFS (months); (**B**) OS (months). Survival probabilities were compared using a two-sided log-rank test.

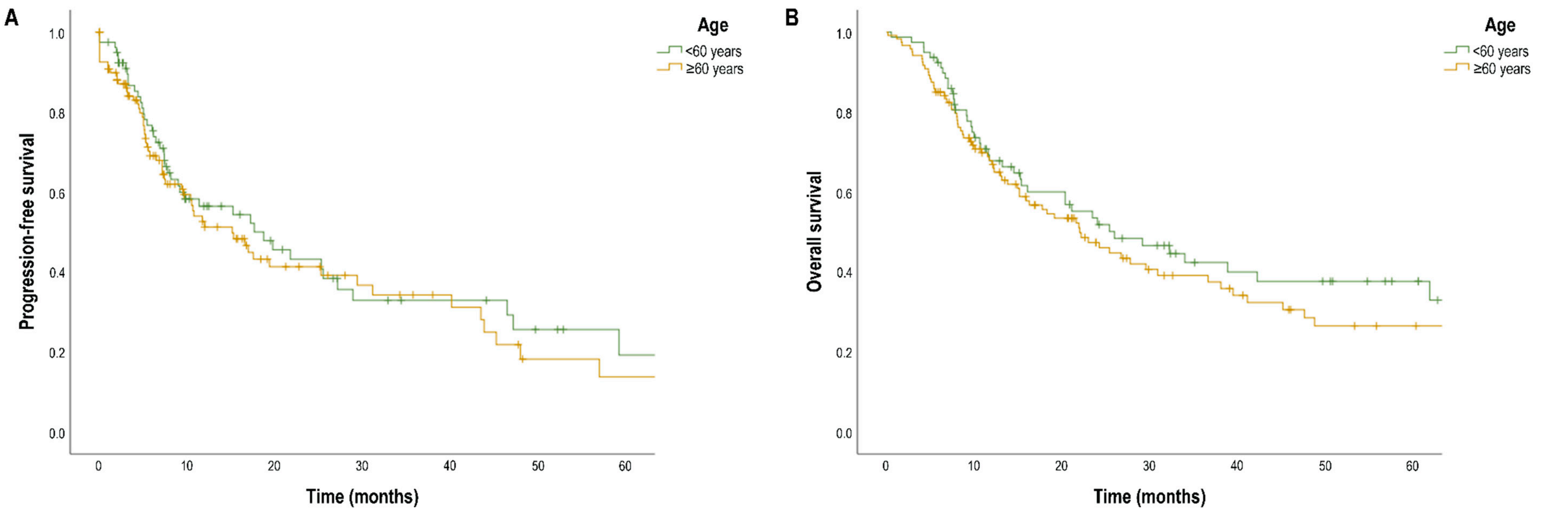

Supplementary Figure S2. Progression-free survival (PFS) and overall survival (OS) in patients with metastatic melanoma treated with BRAF and MEK inhibitors by age group. (**A**) PFS (months) by age group: under 60 years and over 60 years; (**B**) OS (months) by age group: under 60 years and over 60 years. Survival probabilities were compared using a two-sided log-rank test.

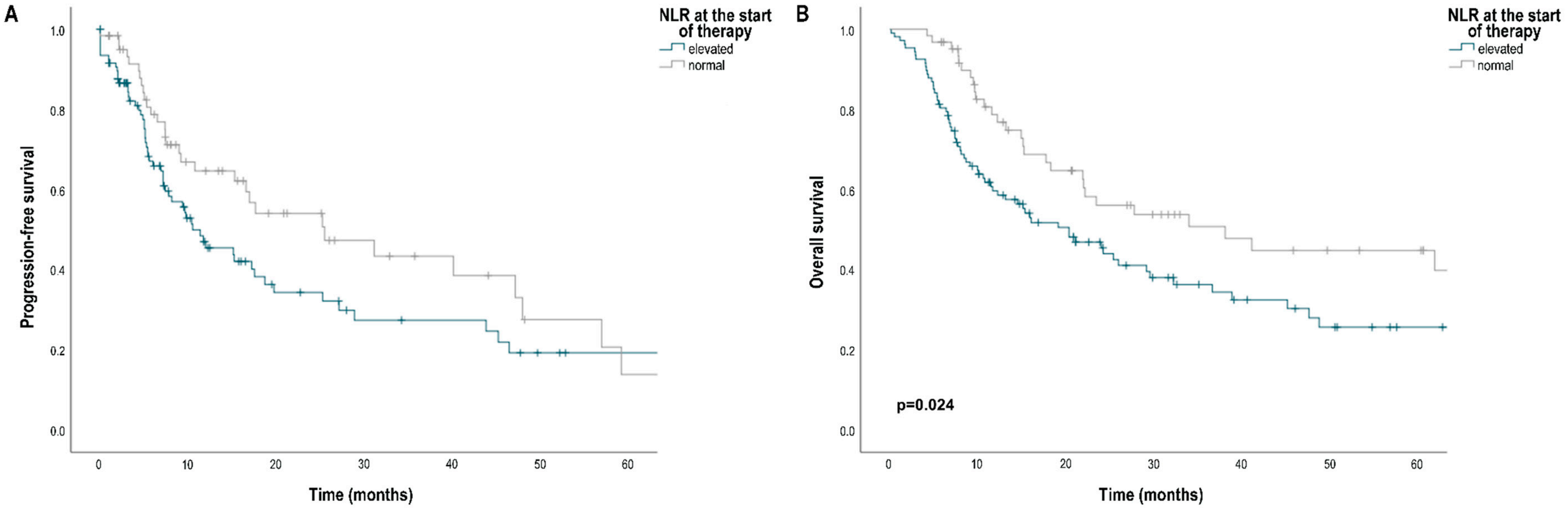

Supplementary Figure S3. Progression-free survival (PFS) and overall survival (OS) in patients with metastatic melanoma treated with BRAF and MEK inhibitors according to baseline peripheral blood NLR. (**A**) PFS (months) according to baseline absolute neutrophil count; (**B**) OS (months) according to baseline absolute neutrophil count. Survival probabilities were compared using a two-sided log-rank test. NLR—neutrophil-to-lymphocyte ratio.

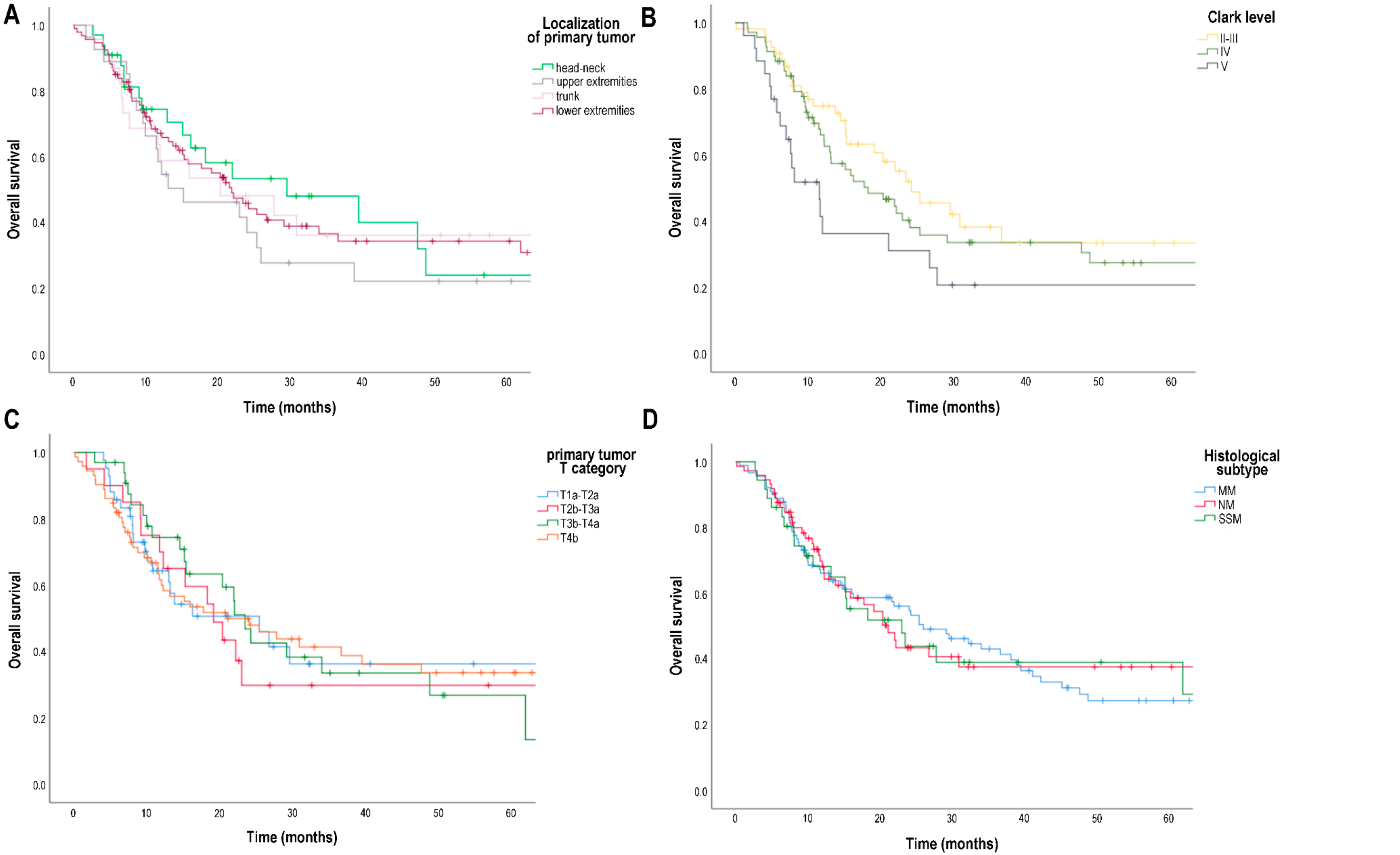
A
Localization of primary tumor
head-neck
upper extremities
trunk
lower extremities
Overall survival
Time (months)
B
Clark level
II-III
IV
V
Overall survival
Time (months)
C
primary tumor T category
T1a-T2a
T2b-T3a
T3b-T4a
T4b
Overall survival
Time (months)
D
Histological subtype
MM
NM
SSM
Overall survival
Time (months)

Supplementary Figure S4. Overall survival (OS) in patients with metastatic melanoma treated with BRAF and MEK inhibitors according to primary tumor localization, Clark invasion level, AJCC 8th edition primary tumor (pT) category and histological subtype. (A) OS (months) according to primary tumor localization (head-neck, upper extremities, lower extremities, trunk); (B) OS (months) according to Clark invasion level (II-III, IV, V); (C) OS (months) according to AJCC 8th edition primary tumor (pT) category (T1a-T2a, T2b-T3a, T3b-T4a, T4b); (D) OS (months) according to primary tumor histological subtype (MM, SSM, NM). AJCC—American Joint Committee on Cancer, MM—unclassified malignant melanoma or no evidence of primary tumor, SSM—superficial spreading melanoma; NM—nodular melanoma. Survival probabilities were compared using a two-sided log-rank test.
